# Supplementary material for: The uptake of key Essential Nutrition Action (ENA) messages and its predictors among mothers of children aged 6–24 months in Southern Ethiopia, 2021: A community-based crossectional study
Source: PLoS One. 2022 Oct 26;17(10):e0275208. doi: 10.1371/journal.pone.0275208 (PMC9604956; doi:10.1371/journal.pone.0275208)
Supplement: S1 File — (DOCX) [file pone.0275208.s002.docx]

## Annex4:--Data collection tool

| Results of questionnaire: - | |
| --- | --- |
| 1. Completed [_____] | 1. Participant Refused [_____] |
| 1. Partially completed [_____] | **4.** |

| Respondents identification ______  Questionnaire Code______  **Instruction: Circle the appropriate answer** provided and where applicable writes the required responses in the spaces provided.  **SECTION 1: m** | | | | | | | | | | | | |
| --- | --- | --- | --- | --- | --- | --- | --- | --- | --- | --- | --- | --- |
| S.N | Questions | | Answers | | | | | | | | | Code |
| 101 | How old are you? Age (in years) | | 1. [___________]  2. I don’t know------------ | | | | | | | | |  |
| 102 | What is your Marital status? | | 1. Married | | | | | | 1. divorced /Separated | | |  |
|  |  |  | 1. un married | | | | | | 1. Widowed | | |  |
| 103 | What is your religion? | | 1. Orthodox | | | | | | 1. Muslim | | |  |
|  |  |  | 1. Catholic | | | | | | 1. Protestant | | |  |
|  |  |  | 1. Other | | | | | |  | | |  |
| 104 | To what ethnicity you belong to? | | 1. Guraghe 2. Amhara  3. Oromo 4. Others. | | | | | | | | |  |
| 105 | What is your educational level? | | 1. No formal education | | | | | 1. 1-8^th^ | | | |  |
|  |  |  | 1. 9-12^th^ | | | | | 1. College and above | | | |  |
| 106 | What is your present Occupation? | | 1. House wife | | | | | 1. Merchant | | | |  |
|  |  |  | 1. Farmer | | | | | 1. Daily laborer | | | |  |
|  |  |  | 1. civil servant | | | | | 1. Others | | | |  |
| 107 | What is the educational level of your husband? | | 1. No formal education | | | | | 1. 1-8^th^ | | | |  |
|  |  |  | 1. 9-12^th^ | | | | | 1. College and above | | | |  |
| 108 | What is his present Occupation? | | 1. farmer | | | | | 2.Government employee | | | |  |
|  |  |  | 3. Merchant | | | | | 4. Daily work | | | |  |
|  |  |  | 5. other | | | | | 6. No work | | | |  |
| 109 | How many members are there within the family?(family size) | | [________________] | | | | | | | | |  |
| 110. | Wealth index measurement | | | | |  | | | | | | Code |
| 1 | Does your household have:(1=Yes, 0=No) | | | | | Electricity | | | | | |  |
|  |  |  |  |  |  | Radio | | | | | |  |
|  |  |  |  |  |  | Television | | | | | |  |
|  |  |  |  |  |  | mobile telephone | | | | | |  |
|  |  |  |  |  |  | A table | | | | | |  |
|  |  |  |  |  |  | A chair | | | | | |  |
|  |  |  |  |  |  | Bed | | | | | |  |
| 2 | Does anyone of your household member have? (1=Yes, 0=No) | | | | | Bicycle | | | | | |  |
|  |  |  |  |  |  | Motorcycle | | | | | |  |
|  |  |  |  |  |  | Bajaj | | | | | |  |
|  |  |  |  |  |  | An animal-drawn cart? | | | | | |  |
|  |  |  |  |  |  | A car/truck? | | | | | |  |
| 3 | Do you have private home?(1=Yes, 0=No) | | | | |  | | | | | |  |
| 4 | Main material of the roof (observe) | | | | | 1. Grass 2. Metal/corrugated iron | | | | | |  |
| 5 | What is the main source of drinking water for your household? (circle or tick on the options) | | | | | 1. Water from spring/ river/ pond 2. Dug well 3. Piped | | | | | |  |
| 6 | What type of fuel does your household mainly use for Cooking?(code based on the respective number) | | | | | 1. Animal dung | | | | | |  |
|  |  |  |  |  |  | 1. wood | | | | | |  |
|  |  |  |  |  |  | 1. Charcoal | | | | | |  |
|  |  |  |  |  |  | 1. Electricity | | | | | |  |
| 7 | Does any member of this household have a bank or microfinance saving account (1=Yes, 0=N0) | | | | |  | | | | | |  |
| 8 | How many of the following animals does the house hold have? (in number) | | | | | Milk Cows | | | | | |  |
|  |  |  |  |  |  | Ox | | | | | |  |
|  |  |  |  |  |  | Hen | | | | | |  |
|  |  |  |  |  |  | Goat/Sheep | | | | | |  |
|  |  |  |  |  |  | Donkey/Horse/Mule | | | | | |  |
| 9 | Does this household own any agricultural land? ( 1=Yes, 0=No) | | | | |  | | | | | |  |
| 10 | How many hectares of agricultural land do members of this household own (in hectares) | | | | |  | | | | | |  |
| 11 | Did you rent/lease out land over the last 12 months? ( 1=Yes, 0=No) | | | | |  | | | | | |  |
| 12 | In the past 12 months how many quintals did you got? [list amount produced for each crop] | | | | | 1. Teff | | | | | |  |
|  |  |  |  |  |  | 2. Barely | | | | | |  |
|  |  |  |  |  |  | 3. Wheat | | | | | |  |
|  |  |  |  |  |  | 4. Maize | | | | | |  |
| 13 | How much is monthly income of the family in birr? | | | | |  | | | | | |  |
| Back ground of the child | | | | | | | | | | | | |
| 111 | Age of the child | | | | | \| 0-6month \| \| --- \| | | | | | |  |
|  |  |  |  |  |  | 2. 7-12month | | | | | |  |
|  |  |  |  |  |  | 3. 13-18month | | | | | |  |
|  |  |  |  |  |  | 4. 19-24month | | | | | |  |
| 112 | Sex of the child | | | | | - - - 1. Male       2. Female | | | | | |  |
| **SECTION 2: MATERNAL HEALTH SERVICE RELATEDCHARACTERSTICS** | | | | | | | | | | | | Code |
| 201 | How many children do you have (parity)? | | | ____________________ | | | | | | | |  |
| 202 | What was your last birth outcome during delivery | | | 1. Still birth 2. Live birth 99. No response | | | | | | | |  |
| 203 | Did you have any history of neonatal death | | | 1. Yes 2. No 99. No response | | | | | | | |  |
| 204 | What seems planning status of your last pregnancy while you got pregnant for the last time? | | | 1. I had a plan and desire to that pregnancy 2. The pregnancy occurred earlier than desired 3. The pregnancy occurred when no or more children were desired | | | | | | | |  |
| 205 | Did you have ANC visit while you were pregnant? | | | 1. Yes 2. No  ______**if no go to 209** | | | | | | | |  |
| 206 | How many times you had got the visit? | | |  | | | | | | | |  |
| 207 | Where was place of your last ANC visit? | | | 1. Health center | | | | 2. hospital | | | |  |
|  |  |  |  | 3.health post | | | | 4.other | | | |  |
| 209 | Where did you give your last birth? | 1. Health center | | | | | 1. Hospital | | | | |  |
|  |  | 1. Health post | | | | | 1. Home | | | | |  |
|  |  | 1. Other specify | | | | |  | | | | |  |
| 210 | In what Mode of delivery you got your child? | 1.spontaneous vaginal delivery | | | | | | | | 2. Instrumental deliver | |  |
|  |  | 3.Caesarean section | | | | | | | |  | |  |
| 301 | How long does it take you to walk to reach nearby health facility from your home? | | | | Minuit/hours [_______]  I don’t know---------------- | | | | | | |  |
|  | What means of transport did you use while you were going to health facility for the last time?(More than one answer is possible) | | | | 1. on foot | | | | | | 1. Vehicles |  |
|  |  |  |  |  | 1. Ambulance | | | | | | 4. Strature |  |
|  |  |  |  |  | 1. Others | | | | | |  |  |
| 302 | Who will decide when you want to go to health facility for maternity services? | | | | 1. Myself | | | | | | 1. Me and my husband |  |
|  |  |  |  |  | 1. My husband | | | | | |  |  |
|  | Did you use maternity waiting room? | | | | 1. yes 2.no if yes skip to | | | | | | |  |
|  | How long you stayed there? | | | | 1.for less than one week  2. one week 3.more than one week | | | | | | |  |
| 303 | Are you member of women health development army (WHDA)? | | | | 1. Yes 2.No | | | | | | |  |
| 304 | Are you model hose hold | | | | 1.yes (assure by observing certificate) 2.No | | | | | | |  |

| **Knowledge of respondents towards key ENA messages** | | | | | |
| --- | --- | --- | --- | --- | --- |
| **1. Knowledge on Exclusive breast feeding** | | | | | |
| Sr no | assessment Knowledge questions on ENA | Response categories | | code | |
|  | Baby should start breastfeeding (If she answers 1) | 1. Immediately after birth 2. Hours after birth 3. Days after birth 4. Don’t Know | |  | |
|  | \| Baby should receive only breast milk \| \| --- \| | 1. From birth to six months 2. Other 3. Don’t know | |  | |
|  | Babies should be given prelacteal feeds | 1. Yes 2. No | |  | |
|  | Colostrum should be fed to the baby | 1. Yes 2. No | |  | |
|  | Babies should feed with which one? | 1. Bottle 2. Cup 3. I don’t know | |  | |
| **2. Knowledge on complementary feeding** | | | | | |
|  | How long after birth a baby should start to receive semi-solid and solid foods?  Age in Months ______________ | 1. At six months 2. Other 3. Don’t know | | |  |
|  | Until what age is it recommended that a mother continues breastfeeding? | 1. Six months or less  2. 6–11 months  3. 12–23 months  4. 24 months and more (correct response) | | |  |
|  | How many times the child should feed solid and/or semi-solid food between per day? If response is not numeric, probe for a numeric response | 1. Number of feedings of solids and/or semi-solid foods _________  2. Don’t know | | |  |
|  | Which one of the following food type your child should get per day?( if she can mention at least 2 | 1. Animal-source foods  2. Pulses and nuts:  3. Vitamin-A-rich fruits and vegetables.)  4. Green leafy vegetables  5. Energy-rich foods | | |  |
|  | Until what age is it recommended that a mother continues breastfeeding?  (24 months and more-correct response) | 1. Six months or less  2. 6–11 months  3. 12–23 months  4. 24 months and more (correct response)  5. Don’t know | | |  |
| **3. Knowledge towards feeding of sick child** | | | | | |
|  | The frequency of breast feeding during and after illness of your baby should be : | 1. More than the usual  2. Same as usual  3. Less than usual  4. Don’t know | | |  |
|  | The amount of fluid offered for sick child during illness should be: | 1. More than the usual  2. Same as usual  3. Less than usual  4. Don’t know | | |  |
|  | The amount of food offered to sick child during and after illness should be: | 1. More than the usual  2. Same as usual  3. Less than usual  4. Don’t know | | |  |
| **4. Knowledge of mothers on nutrition during pregnancy and lactation** | | | | | |
| 14. | How should a pregnant and lactating woman eat in comparison with a non-lactating woman? | 1.Eat more frequently  2. Eat more protein-rich foods  3. Eat more iron-rich foods  4. Use iodized salt when preparing meals  6. Don‟t know | | |  |
| 15. | Most women would benefit from two types of supplements, or tablets, during pregnancy. Which are they? | 1.Iron supplements  2.Folic acid supplements  4.Don‟t know | | |  |
| 16. | What is the health benefit for taking folic acid supplements/tablets? | 1. For normal development of the nervous system of the unborn baby  2. To prevent birth defects  3. Other  4. Don‟t know | | |  |
| **5. Knowledge of mothers on vitamin A deficiency** | | | | | |
| 17. | How can one prevent a lack of vitamin A in the body? | 1. Eat/feed vitamin-A-rich foods – having/giving a diet rich in vitamin A  2. Eat/feed foods fortified with vitamin A  3. Give vitamin A supplements  4. Other  5. Don‟t know | | |  |
| 18. | Mothers should take Vitamin A supplementation as soon as possible (within 45 days) after delivery? | 1. Yes 2. No  3. I don‟t kn | | |  |
| 19. | Can you tell me how you can recognize someone who lacks vitamin A in his or her body?  1 | 1. Weakness/feels less energetic  2. Be more likely to become sick (less immunity to infections)  3. Eye problems: night blindness (inability to see at dusk and in dim light)  4. Other  5. Don‟t know | | |  |
| 20. | Child should take Vitamin A supplementation two times a year after 6 month of age? | 1. Yes 2. No  3. I don‟t know | | |  |
| **6. Knowledge of mothers on prevention of iron deficiency anaemia** | | | | | |
| 21. | Pregnant and lactating mothers should receive iron–folic acid? | | 1. Yes 2. No | |  |
| 22. | What are the health risks for infants and young children of a lack of iron in the diet? | | 1. Delay of mental and physical development  2. Other 3. Don‟t know | |  |
| 23. | Consumption of meat and animal product during pregnancy is helpful to prevent anemia? | | 1. Yes 2. No | |  |
| 24. | Consumption of green leafy vegetables and fruits should be consumed to prevent iron deficiency anemia? | | 1. Yes 2. No | |  |
| **7. Knowledge of mothers on prevention of iodine deficiency** | | | | | |
| 25. | How can iodine deficiency be prevented?  95 | | 1. Eat/prepare foods with iodized salt  2. Other  3. Don’t know | |  |
| 26. | Do you know goiter can be caused by iodine deficiency | | 1. Yes 2. No | |  |
| 27. | When do you think salt should be added in to a stew? | | 1. At the end 2. At the middle 3. At the beginning | |  |
| 28. | Salt hould be stored in dark closed container? | | 1. Ye 2. No | |  |

| **Assessment of practice Towards key ENA messages** | | | |  |
| --- | --- | --- | --- | --- |
| **1. Practice of exclusive breast feeding** | | | | |
| 1 | Have you ever breastfed [NAME]? | | 1. Yes 2. No |  |
| 2 | When did you start breast feed after birth? If less than 1 hour , record immediately(“00” hours),If less than 24 hours, record hours ,Otherwise, record days | | 1. Immediately 2. Hours 3. Days 4. Don’t Know |  |
| 3 | Did you gave sugar water, water, butter, before breast during the first days of the baby’s life? | | 1. Yes 2. No |  |
|  | Did you squeeze out and throw away the first milk (colostrum)? | | 1. Yes 2. No |  |
|  | Did your baby drink anything from a bottle between sunrise yesterday and sunrise today? | | 1. Yes 2. No |  |
| **2.Practice related to Complementary BF** | | | | |
|  | Did you introduce liquids or foods (semi-solid or solid) other than breast milk to the baby? | 1. Yes 2. No if no skip to | |  |
|  | At what age did you first introduce? | 1. 6 months 2. 1 year 3. 2 year 4. Others | |  |
|  | How many times did you feed your child solid and/or semi-solid food between sunrise yesterday and sunrise today? If response is not numeric, probe for a numeric response | 1. Number of feedings of solids and/or semi-solid foods _________  2. Don‟t know | |  |
|  | Was your child breastfed or did he or she consume breastmilk yesterday during the day or at night? | 1. Yes  2. No  3. Don’t know/no answer | |  |
| **3. Practice Feeding of the Sick Child during and after illness** | | | | |
|  | Did your child was sick with in the last two weeks? | 1. Yes 2. No if no Skip to 305 | |  |
|  | How much did you breastfeed your child during and after illness? compared with the usual  (YES=1, others=No) | 1. More  2. Same as usual  3. Less than usual  4. Never gave breast milk | |  |
|  | What amount of fluid did you offered to your child during and after illness? compared with the usual  (YES=1, others=No) | 1. More than the usual  2. Same as usual  3. Less than usual  4. Never gave fluid | |  |
|  | What amount of food did you offered to your child during and after illness? compared with the usual | 1. More than usual  2. Same as usual  3. Less than usual  4. Never gave food | |  |
| **4. Practice of mothers nutrition during pregnancy and lactation** | | | | |
|  | Did you eat one additional meal every day during pregnancy and lactation? | 1. Yes 2. No | |  |
|  | Did you eat a variety of foods, particularly animal products (meat, milk, eggs, etc.), plus fruits & vegetables during pregnancy and lactation? | 1. Yes 2. No | |  |
|  | Did you get iron/folic acid pills to maintain your strength & health during the pregnancy? **Show her or tell the color** | 1. Yes 2. No | |  |
| **5. Practice of mothers on prevention of Vitamin A deficiency** | | | | |
|  | Did you (Mother), take Vitamin A supplementation as soon as possible (within 45 days) after delivery? | 1. Yes 2. No | |  |
|  | Did your child get Vitamin A supplementation two times a year after 6 month of age | 1. Yes 2. No | |  |
|  | I would like to ask you about particular foods you may eat on their own or as part of a dish.  Yesterday, during the day and night, did you eat any of the following foods? | 1. Animal-source foods-Liver, Kidney, Heart, Egg  2. Green vegetables  3. Fruits-Ripe mango, Ripe papaya,  4. I didn‟t eat | |  |
| **6. Practice of mothers on prevention of anemia among mothers and children** | | | | |
|  | Did you (Mothers of infants 0–5 mo.) received iron–folic acid during pregnancy? | 1. Yes 2. No | |  |
|  | Did you eat meat (liver, kidney and heart) and animal product during pregnancy | 1. Yes 2. No | |  |
|  | Did you eat green leafy vegetables and fruits during pregnancy | 1. Yes 2. No | |  |
| **7. Practice of mothers on prevention of iodine deficiency** | | | | |
|  | What kind of salt did you use while you cook family food? (If possible, ask the respondent to show you the salt.) | 1. Iodized  2. Not iodized  3. Don‟t know | |  |
|  | When did you add salt in to the stew? | 1. At the end  2. At the middle  3. At the beginning | |  |
|  | Did you store salt in dark closed container? | 1. Yes 2. No | |  |

**Thank you!!!!**
