# Supplementary material for: The uptake of key Essential Nutrition Action (ENA) messages and its predictors among mothers of children aged 6–24 months in Southern Ethiopia, 2021: A community-based crossectional study
Source: PLoS One. 2022 Oct 26;17(10):e0275208. doi: 10.1371/journal.pone.0275208 (PMC9604956; doi:10.1371/journal.pone.0275208)
Supplement: Attachment — Submitted filename: Comments to the Authors.docx [file pone.0275208.s003.docx]

**General comments**

It is good work. There are a lot of grammar and punctuation error, give time for your document and try to modify them. Above those comments try to see critically your document for minor errors.

**Specific comments**

Title: Import hyphens (Community-based Cross-sectional)

Abstract:

- Be consistent throughout your document (either 6 to 24 months or 6 – 24 months, check with what you put in your title)
- The practice of mothers **towards** key ENA messages…
- --- the magnitude of good ENA practice was **296.** (47.4%)—better to remove the figure and put only the percentage in the abstract
- Mother’s education level [AOR: 3.90, 95%CI: 1.79, 8.51], parity [AOR: 2.72, 95%CI: 1.51,4.89], place of delivery[AOR: 2.75, 95%CI: 1.17,6.49], having PNC service[AOR: 2.95, 95%CI: 1.91, 4.57] and being a model household [AOR: 3.83,95%CI: 2.58, 5.69] were identified as a significant determinants of a good practice---- Better if you put separately which are positively associated, and which are negatively associated.
- put a space between 95%CI---95% CI, a space between word and [
- The overall practice of key ENA messages in the study area was low…. Compared to what?
- The MNCH, SDS, PNC--- if possible try to avoid abbreviations in Abstract, unless you have to state them initially
- Key messages – better if you include your study area, and study participants

**Background**

- -----programs, and strategies[1]—Put a space between words and references across the whole document ( strategies [1])
- The key areas of ENA is in children between 6 – 23 months but your study is on 6 – 24 months, why?
- Why you use the report of EDHS 2016 ?
- Your statement of the problem states around, not exactly on the ENA components. And you did not clearly identify studies on ENA, and the limitations of each study, and what your study adds on top of those studies (the gap seems vague).

**Methods and materials**

- NGO ----state it
- The study populations were women from selected kebeles who fulfilled the eligibility criteria – this is totally wrong. Clearly put the difference between your source population and study population. Then you have to put the exclusion and inclusion criteria separately
- The sampling procedure you followed to get the study participants is not feasible in such community (Community which is dispersed randomly, and have no household number) … in such community it is better to use cluster sampling technique.
- Why nurses as data collector, and PH as supervisor? Why not other professionals too
- The variance inflation factor(VIF) >10 was used to check for multicollinearity between the explanatory variables---there is no space in logistic regression to run multicollinearity, this kind of test seems pseudo, better if you see the standard errors of each variable.
- Finally, the regression analysis’ findings have been reported using **odds ratios** and the corresponding 95 % confidence interval --- Adjusted odds ratios
- Did you check model fitness and model adequacy, Did you check outliers…it should be mentioned
- Ethical clearance was obtained from the Ethical Review Committee of Wachemo University School of Public Health, College of Medicine and Health Science—Put the IRB number

**Result**

- Where did you get such like Age classification of children ---- 6-11, 12-17, 18-23
- Family size classification –why you use cut of 5, what is the recommendation in Ethiopian context
- **When it came to the number of children**, 293(47.0%) of respondents were multiparous. Do you mean children are multiparous???
- Remove the words like the majority, minority, small percentage….. they have no common interpretation
- For numbers >99 it is advisable to put in number rather than stating in words
- The font size across your document should be similar
- Your discussion flow should be from your study finding, then to similar study settings, then to Africa, then to the world. But you didn’t follow coherence.
- Make your discussions short and smart, it seems the report of your and other findings
- Your justifications in the discussion should be supported by some evidence, even if your thought of thinking may be there but advisable to support with references
- Your conclusion should be strong enough based on standard’s

References

- Check the grey literatures when you use ENDNOTE, they need manual arrangement in the software
