# Supplementary material for: The uptake of key Essential Nutrition Action (ENA) messages and its predictors among mothers of children aged 6–24 months in Southern Ethiopia, 2021: A community-based crossectional study
Source: PLoS One. 2022 Oct 26;17(10):e0275208. doi: 10.1371/journal.pone.0275208 (PMC9604956; doi:10.1371/journal.pone.0275208)
Supplement: Attachment — Submitted filename: Response to Reviewers.docx [file pone.0275208.s004.docx]

**AUTHORS’ RESPONSE TO ACADEMIC EDITOR AND REVIEWERS**

1. **AUTHOR’S RESPONSE TO EDITOR**

**Dear: Joseph Donlan (Academic Editor)**

We thank you for a thorough reading and constructive comments and suggestions on our manuscript and for the opportunity to revise and resubmit. We are pleased to submit the revised research article “**The uptake of key essential nutrition action (ENA) messages and its predictors among mothers of children aged from 6-24months in Southern Ethiopia, 2021: A community-based crossectional study**” for your consideration in the special collection of Plos One. On the following pages, you will find our responses to all the three reviewers' comments. On behalf of my co-authors, I thank you for your consideration of this resubmission. We appreciate your time and look forward to your response.

Sincerely,

Aklilu Habte(MPH)(corresponding author)

[**aklilihabte57@gmail.com**](mailto:aklilihabte57@gmail.com)

**Dear Dr. Habte,**

Thank you for submitting your manuscript to PLOS ONE. After careful consideration, we feel that it has merit but does not fully meet PLOS ONE’s publication criteria as it currently stands. Therefore, we invite you to submit a revised version of the manuscript that addresses the points raised during the review process.

Your manuscript has been assessed by two expert reviewers, whose comments are appended below. The reviewers have highlighted concerns about several aspects of the methodology and presentation which must be addressed for the manuscript to be suitable for publication. Please ensure you respond to each point carefully in your response to reviewers document, and modify your manuscript accordingly.

**Response:** thank you for giving us the chance to revise our work by incorporating all valuable comments and suggestions raised by the reviewers.

**Journal Requirements:**

When submitting your revision, we need you to address these additional requirements.

1. Please ensure that your manuscript meets PLOS ONE's style requirements, including those for file naming. The PLOS ONE style templates can be found at

<https://journals.plos.org/plosone/s/file?id=wjVg/PLOSOne_formatting_sample_main_body.pdf> and

<https://journals.plos.org/plosone/s/file?id=ba62/PLOSOne_formatting_sample_title_authors_affiliations.pdf>

**Response:** Thank you for your suggestion. We have submitted the manuscript as per the journal requirement.

2. Please review your reference list to ensure that it is complete and correct. If you have cited papers that have been retracted, please include the rationale for doing so in the manuscript text, or remove these references and replace them with relevant current references. Any changes to the reference list should be mentioned in the rebuttal letter that accompanies your revised manuscript. If you need to cite a retracted article, indicate the article’s retracted status in the References list and also include a citation and full reference for the retraction notice.

**Response:** Thank you for recommending that we have checked the appropriateness of the references we used in the manuscript. We reviewed all of the references as per your suggestion, and none of them have retracted references.

_____________________________END________________________________________

THANK YOU!!!

**AUTHORS’ RESPONSE TO REVIEWERS**

**Dear: Reviewers**

We thank you for a thorough reading and constructive comments and suggestions on our manuscript and for the opportunity to revise and resubmit. We are pleased to submit the improved research article **“The uptake of key essential nutrition action (ENA) messages and its predictors among mothers of children aged from 6-24months in Southern Ethiopia, 2021: A community-based crossectional study”** for your consideration in the special collection of Plos One. On the following pages, you will find our response to the reviewers’ comments. On behalf of my co-authors, I thank you for your consideration of this resubmission. We appreciate your time and look forward to your response independently as “Authors’ response to Reviewer#1”, and “Authors’ response to Reviewer#2”.

Sincerely,

Aklilu H. (MPH)(corresponding author)

[**aklilihabte57@gmail.com**](mailto:aklilihabte57@gmail.com)

**Authors’ Response to Reviewer**#**1(Dear Getahun Fentaw Mulaw)**

**General comments**

It is good work. There are a lot of grammar and punctuation error, give time for your document and try to modify them. Above those comments try to see critically your document for minor errors.

**Response:** Thank you very much for taking the time to review our work and for your positive feedback. We got all your constructive comments and suggestions as valuable input for the readability of our work and we have tried to respond as follows. We give a due emphasis to those grammatical and Punctuation errors by involving experts in language and literature and we have tried to correct them in the revised version of the manuscript.

**Specific comments**

**Comment1:** Import hyphens (Community-based Cross-sectional)

**Response:** Thank you for your comment we have corrected and highlighted it on the title page of the “Revised manuscript with track changes”, Line3, Page1.

**Comment2:** Be consistent throughout your document (either 6 to 24 months or 6 – 24 months, check with what you put in your title)

**Response:** We become consistent with “6-24 months” throughout the manuscript.

**Comment3:** the magnitude of good ENA practice was 296. (47.4%)—better to remove the figure and put only the percentage in the abstract

**Response:** Thank you for the meticulous review of the manuscript. We have corrected and highlighted it in the “Abstract” section of the “Revised manuscript with track changes”, Line46, Page2.

**Comment4:** Mother’s education level [AOR: 3.90, 95%CI: 1.79, 8.51], parity [AOR: 2.72, 95%CI: 1.51,4.89], place of delivery[AOR: 2.75, 95%CI: 1.17,6.49], having PNC service[AOR: 2.95, 95%CI: 1.91, 4.57] and being a model household [AOR: 3.83,95%CI: 2.58, 5.69] were identified as a significant determinants of a good practice---- Better if you put separately which are positively associated, and which are negatively associated.

**Response:** thank you so much. As per your suggestion, narrate the predictors and the corrected statement is highlighted in the “Abstract” section of the “Revised manuscript with track changes”, Line52-53, Page2.

**Comment5:** Put a space between 95%CI---95% CI, a space between word

**Response:** We appreciate your in-depth review of our work. As per suggestion we have corrected the typological error and highlighted them in the “Abstract” section of the “Revised manuscript with track changes”, Line49-53, Page2.

**Comment6:** The MNCH, SDS, PNC--- if possible try to avoid abbreviations in Abstract, unless you have to state them initially

**Response:** We removed all of the abbreviations and wrote them in full form and highlighted them in the “Abstract” section of the “Revised manuscript with track changes”

**Comment7:** Keywords – better if you include your study area, and study participants **Response:** we included some additional keywords, as per your suggestion.

**Comment8:** -----programs, and strategies[1]—Put a space between words and references across the whole document ( strategies [1])

**Response:** We appreciate your meticulous review. We follow your suggestion throughout the manuscript and the amendments were highlighted in the “Revised manuscript with track changes”.

**Comment10:** Why did you use the report of EDHS 2016 ?

**Response:** thank you for asking. We use the 2016 EDHS report on the level of chronic malnutrition (Stunting) to show the severity of the problem which is mainly linked to the low uptake of Key ENA messages.

**Comment12:** Your statement of the problem states around, not exactly on the ENA components. And you did not identify studies on ENA, and the limitations of each study, and what your study adds on top of those studies (the gap seems vague).

**Response:** thank you very much for your crucial comment. Of course in the initial version of the manuscript, we failed to address the gap that might be addressed by the current study. As per your suggestion, we tried to show the limitations of the previously conducted study and the strength of the current study. The added statements were highlighted in the “Background” section of “Revised manuscript with track changes”, Line111-117, Page4.

**Comment13:** NGO ----state it

**Response:** we stated and highlighted it in the “Study area, design, and period” section of “Revised manuscript with track changes”, Line129, Page5.

**Comment14:** The study populations were women from selected kebeles who fulfilled the eligibility criteria – this is totally wrong. Clearly put the difference between your source population and study population. Then you have to put the exclusion and inclusion criteria separately

**Response:** We appreciate your insightful comments regarding the population of the study. Of course, we made it too ambiguous for the readers to understand, and we have corrected it as per your comment and highlighted it in the “Populations of the study” section of “Revised manuscript with track changes”, Line132-133, Page5.

**Comment15:** The sampling procedure you followed to get the study participants is not feasible in such community (Community which is dispersed randomly, and have no household number) … in such community it is better to use cluster sampling technique.

**Response:** Thank you for your comments and suggestion. Of course, that might be difficult and raise the issue of feasibility. But the implementation of a community health information system (CHIS) at the kebele level made the procedure easy and we preferred that. Initially, we selected those households with eligible study participants. Then we gave a code to those Households and we made a sampling frame. In this way, it was feasible for the data collectors and to access the study participants.

**Comment16:** Why nurses as data collector, and PH as supervisor? Why not other professionals too

**Response:** Nurses outnumber other health professionals in the study area, and they participated in several data collection activities and were relatively highly experienced. As a result, we were intrigued by them. Public Health officers, on the other hand, were involved in a variety of supervisory activities, and we used them as such.

**Comment17:** The variance inflation factor(VIF) >10 was used to check for multicollinearity between the explanatory variables---there is no space in logistic regression to run multicollinearity, this kind of test seems pseudo, better if you see the standard errors of each variable.

**Response:** Thank you for your insightful comment and suggestion, which allows us to include what we missed to report but we did. Accordingly, we remove the unnecessary ones and add the requirements that you suggested to us. The added statements were highlighted in the “Data analysis” section of “Revised manuscript with track changes”, Line197-200, Page7.

**Comment18:** Finally, the regression analysis’ findings have been reported using odds ratios and the corresponding 95 % confidence interval --- Adjusted odds ratios

**Response:** We have corrected and highlighted it in the “Data analysis” section of “Revised manuscript with track changes”, Line195, Page7.

**Comment19:** Did you check outliers…it should be mentioned

**Response:** yes we have checked outliers by running histogram and box plot and we didn’t find out outliers.

**Comment20:** Ethical clearance was obtained from the Ethical Review Committee of Wachemo University School of Public Health, College of Medicine and Health Science—Put the IRB number

**Response:** thank you for the reminder. As per your suggestions we have added the IRB number and highlited it in the “Ethical approval and consent to participate” section of “Revised manuscript with track changes”, Line245, Page8.

**Comment21:** Where did you get such like Age classification of children ---- 6-11, 12-17, 18-23

**Response:** Thank you for asking. Our reference for the categorization was a study conducted in the ENA package in Northern Ethiopia (Reference no. 24).

**Comment22:** Family size classification –why do you use a cut of 5, what is the recommendation in the Ethiopian context

**Response:** Thank you for inquiring. The majority of studies in Ethiopia utilize 5 as the cut-off point for family size, which is the nearest to the national total fertility rate.

**Comment23:** When it came to the number of children, 293(47.0%) of respondents were multiparous. Do you mean children are multiparous???

**Response:** it was referring to the mothers and we have corrected and highlighted it in the “Maternal and child health service-related characteristics” of the “Revised manuscript with track changes”, Line262, Page10.

**Comment24:** Remove the words like the majority, minority, small percentage….. they have no common interpretation

**Response:** thank you for your meticulousious review. To minimize ambegiousity for the readers we replaced those approximation terms with the actual figures throughout the results.

**Comment24:** The font size across your document should be similar

**Response:** thank you for the comment. Regarding the font sizes, we followed the journal guide line throughout the manuscript. According to the submission guide lineThe main titles should be put in first in heading 1 with a font size of 18 and the sub headings with heading 2 and font size of 16. . <https://journals.plos.org/plosone/s/file?id=wjVg/PLOSOne_formatting_sample_main_body.pdf>

**Comment26:** Your discussion flow should be from your study finding, then to similar study settings, then to Africa, then to the world. But you didn’t follow coherence. Make your discussions short and smart, it seems the report of your and other findings

**Response:** We appreciate your thorough review of the manuscript, and we regard your suggestion being an important contribution to improving the readability of our work. As per your suggestion, we reduced some of the unnecessary statements which were out of the objectives of the study and we make the discussion section relatively short and precise. We have now rectified the chronological order. Those ideas that were beyond the study's objectives but addressed in the discussion section were now removed.

**Comment27:** Your conclusion should be strong enough based on standard’s

**Response:** We have tried to make the conclusion more stringent based on the findings of the study.

**Comment28:** Check the grey literature when you use ENDNOTE, they need manual arrangement in the software

**Response:** thank you for allowing us to revise the references that we have used throughout the manuscript. Of course, we got some formatting errors and we have corrected them in the revised version of the manuscript.

Finally, we want to appreciate you for your constructive and insightful comments and suggestions on the manuscript.

__________________________________________________________________________________________________________END________________________________________

THANK YOU!!!

**Authors’ Response To Reviewer**#**2( Dear Christian Bwangandu Ngandu)**

**General Comment:** Five predictors (maternal, household) for essential nutrition message for nutrition of children before 1000 days of life have been set up with the manuscript. Individual, maternal, household and health system factors need more focus for improving child nutrition and health by policy-makers.

The manuscript reads well with consistency between sections although the wordcount remains wide. I appreciated the background and methods sections, they look very informative and consistent with the title and the aim of the study. Some restructuration and editing need to be done in the results, discussion, and reference list for better understanding of the text. The discussion is long as the amount of data that the paper has gathered, analyzed and interpreted. Also, the limitations of the study seem unclear, and some contrasting results have to be clarified and detailed.

**Response:** Thank you very much for taking the time to review our work and for your positive feedback. We received your thoughtful review, along with helpful feedback and suggestions, as a valuable contribution to our ongoing work. We've attempted to respond to your comments and suggestions as follows.

**Commnet1:** When two stages sampling methods for participants has been considered, the authors should clarify and explain the process. Kebele level with simple random selection, household level with random selection, and participants level with simple random selection look like three stages of sampling instead of two.

**Response:** We appreciate your meticulous review of the manuscript. We accept your comment and we replaced the term “two-stage sampling” with “a multi-stage sampling” technique. The amendment was highlighted in the “Sampling procedures” section of the “Revised manuscript with track changes”, Line142, Page5.

**Comment2:** When the dates are mentioned in the methods section, the date doesn’t add value to the title of each table.

**Response:** as per your suggestion we removed the redundant dates from the titles of each table. The corrected version is highlighted in the titles of all the tables.

**Comment3:** Knowledge of respondent section: the sentence should start by a capital letter.

**Response:** we corrected and highlighted in Line277, Page12 of the “Revised manuscript with track changes”

**Comment4:** confusing results come out from the association between being primiparous mothers and good ENA messages because of the free time to care for children and to cook healthy food for the family. But this is a bit confusing and contrasting with other studies where being multiparous could enable mothers to keep more knowledge and experienced practices compared to primiparous mothers. How the authors can discuss these explanations?

Response: thank you for your meticulous review. Of course, almost all studies showed a positive association between ENA practices and being multiparous. This make us to re-examine and evaluate our data set and we got that we have made a mistake during coding and categorizing of the variable, parity. When we re-run the analysis, we got the variable primiparous as a preventive factor for a good uptake of ENA. Accordingly, we have changed the statement throughout the manuscript. The correct statements and figures were highlighted in the:

- “Abstract” section, Line53, Page2
- “Table5”
- Line 353-355, Page15, and
- “Discussion” section, Line 431-441, Page 19 the “Revised manuscript with track changes”

**Comment5:** Multivariable table 5 shows the significant variables that are associated with good practices of key ENA in the study. Being educated (more than secondary level), birth at health facilities, prenatal care (more than four time). The authors should show the variables that have been removed from the adjusted models because of the collinearity.

**Response:** as we tried to indicate in the “Data analysis” section of the revised “Revised manuscript with track changes”, the level of multicollinearity was examined using standard error cut-off two, but no multicollinearity was found among the variables studied. So we didn’t have any reason to remove those variables from the model and we reported them in the current form.

**Comment6:** Limitations of the study and bias (recall bias and desirability bias) has been risen in the study. The authors should say how they attempt to mitigate this risk of bias?

Response: We appreciate your in-depth review. We have added all the efforts that we have made to minimize a recall bias in the “Data quality management” section of the revised “Revised manuscript with track changes”, Line173-176, Page6.

Comment6: regarding the references

6.1: Check for the use of capital letters in titles, abbreviation for the journal, the consistency of page numbers in the references.

6.2. The use of capital letter in the titles (either for the first letter of the title or first letter for each word) should be consistent for the whole list of references.

The consistency needs to be followed with the style of references, particularly the using of “et al”. For instance, this term should be used with consistency after the third authors when the list of authors includes more than six authors. See references number 6, 15,19, 25…

• The authors should edit the spelling of the WHO as author of the citation in the list of references: either writing “WHO”, or World Health Organization” instead of “Organization, WH”. See references number 1, 2, 8, 22, 26…

**Response:** thank you for the in-depth review, which allows us to revise all the references that we have used throughout the work. As per your recommendation, we have corrected all the references accordingly by using a Vancouver style. Beyond that, we also found some redundant references and now all were addressed.

__________________________________________________________________________________________________________END________________________________________

THANK YOU!!!
