# Supplementary material for: The uptake of key Essential Nutrition Action (ENA) messages and its predictors among mothers of children aged 6–24 months in Southern Ethiopia, 2021: A community-based crossectional study
Source: PLoS One. 2022 Oct 26;17(10):e0275208. doi: 10.1371/journal.pone.0275208 (PMC9604956; doi:10.1371/journal.pone.0275208)
Supplement: Attachment — Submitted filename: Response to Academic Editor.docx [file pone.0275208.s005.docx]

**AUTHORS’ RESPONSE TO ACADEMIC EDITOR AND REVIEWERS**

1. **AUTHOR’S RESPONSE TO EDITOR**

**Dear: Saqlain Raza (Academic Editor)**

We thank you for a thorough reading and constructive comments and suggestions on our manuscript and for the opportunity to revise and resubmit. We are pleased to submit the revised research article “**The uptake of key essential nutrition action (ENA) messages and its predictors among mothers of children aged from 6-24months in Southern Ethiopia, 2021: A community-based crossectional study**” for your consideration in the special collection of Plos One. On the following pages, you will find our responses to all the three reviewers' comments. On behalf of my co-authors, I thank you for your consideration of this resubmission. We appreciate your time and look forward to your response.

Sincerely,

Aklilu Habte(MPH)(corresponding author)

[**aklilihabte57@gmail.com**](mailto:aklilihabte57@gmail.com)

**Comment 1:** In Table 1, the variable 'age' has been categorized as categorical variable, and estimated its frequencies and percentages of age-groups. Secondly, in the results before Table 1, authors found mean and SD of age. This creates ambiguity of whether the variable 'age' has been considered as continuous of categorical. Generally, we take the variable 'age' as continuous variable. Authors need to follow the variable type and justify the analysis and results in the same way.

**Response:** We appreciate your inquiry. The first statement that mentioned age in terms of mean and standard deviation was to show the population's overall age, as mentioned by almost all previous studies. presenting the overall age distribution of the study population is found to be helpful to assess the general picture of the population as a background characteristics. However, to run a logistic regression analysis, we convert the continuous variable age into a categorical variable (by age groups).

**Comment2:** In Table 1, authors have estimated WIQs along with socioeconomic status of the participants. It is quite a bit confusing numbers in the frequency. Authors need to ensure the authenticity of the calculations done through PCA.

**Response:** we appreciate your comment. We have discussed the way of principal component analysis and highlighted it in the “Data analysis” section of the “Revised manuscript with track changes”, Line179-187, Pages 6. The gross frequencies were nearly identical since the component with maximum variation was equally divided into quintiles but the variation was observed in the distribution of study participants’ wealth index across the outcome variable, as highlighted in Table 5.

**Comment3:** Standardized age-groups for children are 6-11 months, 12-17 months, and 18-23 months. 23 months means the child has completed 23 months or 2 years of age. Authors can adjust children according to standardized format.

**Response:** thank you for your valuable suggestion. We have corrected it as per your suggestion and highlighted it in “Table1” of the “Revised manuscript with track changes”, page 9.

Comment 4: In Table 3, only 63 children were not exclusively breastfed. In another variable on the same table, 139 children were given some food other than breast milk. The two variables contradicts each other. This is a question mark on the data quality. How would the authors justify this?

**Response:** Thank you very much for your thorough review, which allowed us to re-analyze our data set and avoid sharing incorrect findings with the readers. Now we corrected it and highlighted it in “Table 3” of the “Revised manuscript with track changes”, page 12

**Comment 5:** In Table 5, give only one of 'COR' or 'AOR' and remove the other.

**Response:** We removed the column containing the crude odds ratio (COR) and only display the adjusted odds ratio (AOR) as shown by “**Table 5**” of the “Revised manuscript with track changes”, page 16

**Comment 6:** Include in your study some more relevant literature like:

<https://journals.plos.org/plosone/article?id=10.1371/journal.pone.0263470>

<https://pubmed.ncbi.nlm.nih.gov/35211439/>

**Response:** thank you for your suggestion. We got both publications as a valuable input for the manuscript and we cited the accordingly as reference 12 and 24 in the Revised manuscript.

**Comment7:** In the abstract, methods section is unnecessarily lengthy. Authors need to shorten it.

**Response:** we appreciate you suggestion and we corrected it as per your suggestion. The corrected verion id highlighted in the “Abstract” section of the “Revised manuscript with track changes”, Line34-40, page 2

_____________________________END________________________________________

THANK YOU!!!
